# Vector Flow Imaging for Carotid Artery Stenosis Assessment: A Scoping Review

**DOI:** 10.3390/diagnostics16172713

**Published:** 2026-08-25

**Authors:** Alexander Cuculiza Henriksen, Rikke Baarts, Nathalie Sarup Panduro, Emma Kanchana Ertner Bengtsson, Dennis Zetner, Lars Lönn, Charlotte Mehlin Sørensen, Jørgen Arendt Jensen, Michael Bachmann Nielsen

**Affiliations:** 1 Department of Diagnostic Radiology, Copenhagen University Hospital Rigshospitalet, 2100 Copenhagen, Denmark; 2 Department of Clinical Medicine, University of Copenhagen, 2200 Copenhagen, Denmark; 3 Department of Biomedical Sciences, University of Copenhagen, 2200 Copenhagen, Denmark; 4 Center for Fast Ultrasound Imaging, Department of Health Technology, Technical University of Denmark, 2800 Lyngby, Denmark

**Keywords:** vector flow imaging, carotid artery stenosis, ultrasound, hemodynamics, wall shear stress, scoping review, diagnostic imaging

## Abstract

**Background:** Conventional Doppler ultrasound is limited by its angle dependence and its inability to characterize complex flow patterns adequately. Vector flow imaging (VFI) is an angle-independent ultrasound technique that enables two-dimensional velocity vector mapping and the assessment of hemodynamic parameters. This scoping review aims to map the current evidence on the application and validation of VFI for assessing carotid artery stenosis (CAS). **Methods:** Using the Population–Concept–Context framework, we included studies that evaluated the application, validity, or outcomes of vector flow imaging in individuals with suspected or confirmed carotid artery stenosis, as well as in healthy controls used for comparison or validation, in clinical or research settings. Five electronic databases were searched from inception through 30 April 2026. Studies limited to conventional Doppler ultrasound or to animal or phantom models without human validation were excluded. Data were synthesized using descriptive numerical analysis and narrative thematic synthesis. **Results:** A total of 48 sources of evidence met the inclusion criteria. Six trial registrations without reported results were excluded from synthesis, leaving 42 synthesized sources, including 40 original studies and two reviews. The most frequently reported parameters were wall shear stress (*n* = 25), turbulence indices (*n* = 11), and velocity vectors (*n* = 14). Most studies were cross-sectional (*n* = 20) or technical validation studies (*n* = 13) with small sample sizes (median 31 participants, IQR 10.5–57; range 1–476). The dominant research focus was plaque vulnerability assessment, while fewer studies addressed stenosis grading. Only eight studies reported diagnostic-performance metrics; none employed prespecified thresholds, two reported blinding, and none adhered to the Standards for Reporting Diagnostic Accuracy Studies guidelines (STARD). **Conclusions:** VFI enables detailed characterization of carotid hemodynamics, but the available evidence remains preliminary and does not establish clinical utility. Standardized, adequately powered studies with external validation are required before clinical implementation.

## 1. Introduction

Carotid artery stenosis (CAS) accounts for approximately 8–15% of ischemic strokes and transient ischemic attacks [1,2]. Accurate assessment of CAS is essential for risk stratification and treatment decision-making, particularly in determining candidacy for carotid endarterectomy or carotid artery stenting, as current clinical guidelines rely on stenosis severity, symptom status, and patient characteristics to guide intervention decisions [1,2,3]. Multiple imaging modalities are used to assess CAS, including computed tomography angiography (CTA), magnetic resonance angiography (MRA), digital subtraction angiography (DSA), and Doppler ultrasound [1,4]. While CTA, MRA, and DSA provide anatomical visualization of stenosis, Doppler ultrasound offers the advantages of being non-invasive, portable, radiation-free, and cost-effective [5,6,7]. However, expert consensus recommends caution when using a single Doppler examination as the sole diagnostic method [1,4]. Conventional Doppler has important limitations, including angle-dependent velocity measurements, inability to visualize complex flow patterns, and obstacles in accurately assessing stenosis severity when flow is tortuous or optimal insonation angles cannot be achieved.

Vector flow imaging (VFI) is an angle-independent ultrasound technique that enables two-dimensional velocity vector mapping, overcoming the angle dependency that limits conventional Doppler. VFI is now available on some clinical ultrasound systems [8,9,10,11]. It can be conducted using various methods: transverse oscillation [12,13], speckle tracking [14], plane wave imaging with velocity vector estimation [15,16,17], synthetic aperture techniques [18,19], and multidirectional Doppler-based vector estimation approaches, including vector projectile imaging [20,21]. This capability enables the visualization of complex flow patterns and the quantification of parameters not accessible with conventional Doppler, including wall shear stress, turbulence indices, and vortex formation [22,23]. Despite these theoretical advantages and commercial availability, the evidence base for VFI’s clinical application in CAS assessment remains limited. The aim of this scoping review was to critically review the current evidence regarding the use of VFI in CAS assessment and evaluate its potential role in clinical practice.

## 2. Materials and Methods

### 2.1. Review Methodology

This scoping review was conducted in accordance with the Joanna Briggs Institute methodology for scoping reviews [24] and reported in accordance with the Preferred Reporting Items for Systematic Reviews and Meta-Analyses extension for Scoping Reviews (PRISMA-ScR) guidelines. A checklist is available in Appendix A [25]. The review followed Arksey and O’Malley’s five-stage framework [26], as refined by Levac et al. [27]: (1) identifying the research question; (2) identifying relevant studies; (3) study selection; (4) charting the data; and (5) collating, summarizing, and reporting results.

### 2.2. Protocol

A protocol for this review was developed a priori and registered on the Open Science Framework (https://osf.io/68ua7 (accessed on 20 August 2026); DOI: 10.17605/OSF.IO/68UA7). The protocol specified the research questions, inclusion and exclusion criteria, search strategy, and data extraction plan. Deviations and their justifications are detailed in Appendix B.

### 2.3. Research Questions and Inclusion and Exclusion Criteria

The primary research question was as follows: What is the scope and nature of the evidence regarding the use of VFI for the assessment of CAS? Three sub-questions addressed the application of VFI in clinical evaluation, the hemodynamic insights VFI offers compared to other imaging modalities, and the clinical outcomes reported.

Inclusion criteria:

The inclusion criteria were defined using the Population–Concept–Context framework and addressed patients diagnosed with CAS (population), the application, validity, and outcomes associated with VFI (concept), and clinical or research settings where VFI is used to assess stenosis (context):

- Participants/Population: We included studies involving human participants with suspected or confirmed CAS, including both symptomatic and asymptomatic individuals. Studies involving healthy controls, when used for comparison or validation purposes, were also included. All ages, genders, ethnicities, and any degree of stenosis severity were included.
- Concept: We included studies examining VFI technology for carotid artery assessment. VFI was defined as angle-independent ultrasound techniques, including transverse oscillation, plane wave imaging with velocity vector estimation, and synthetic aperture techniques with directional flow capability. Studies examining VFI-derived parameters, including two-dimensional velocity vector maps, flow patterns, vortex visualization, volume flow measurements, wall shear stress estimation, velocity magnitude and direction, and complex flow characterization, were included.
- Context: We included studies conducted in clinical settings (hospitals, outpatient clinics, and vascular laboratories) or research settings (validation studies, proof-of-concept studies, and comparative studies) in any geographic location.

All research study designs were eligible. Conference abstracts reporting original data with sufficient methodological detail and theses/dissertations were also included.

Studies were eligible regardless of publication language, provided they were indexed in international databases with English-language abstracts. Full-text translation for these studies was performed using AI-assisted translation (Claude Code v2.1.30, Anthropic) to enable data extraction and quality assessment. All extracted methodological details, numerical results, and table values were independently verified against the original-language full texts by reviewers proficient in the relevant languages. There was no restriction on publication date.

Exclusion criteria

Studies were excluded if they examined only conventional Doppler ultrasound without VFI technology; only color Doppler or power Doppler without directional velocity vector capability; or exclusively arteries other than the carotid without carotid artery data or animal, phantom, computational, or in vitro models without human carotid validation.

### 2.4. Information Sources and Search Strategy

A systematic search was conducted across five electronic databases (MEDLINE via PubMed, Embase via Ovid, Scopus, Web of Science, and IEEE Xplore) and one trial registry (the WHO International Clinical Trials Registry Platform) from database inception through 30 April 2026. Searches were supplemented by reference list screening and forward citation searching of included studies. The search strategy was developed in collaboration with an experienced medical librarian and combined two main concepts: CAS and vector flow imaging technology. The original search strategy was revised to include studies with carotid plaques and other techniques potentially related to VFI.

Complete search strategies for all databases are provided in Appendix C.

### 2.5. Selection of Sources of Evidence and Data Charting

Two authors conducted study selection in the Covidence systematic review software using a two-stage screening process. Titles and abstracts were screened independently, followed by independent full-text assessment against the complete eligibility criteria. Disagreements at either stage were resolved through discussion, with consultation of a third author when necessary.

A custom-built database incorporating a standardized data charting form was developed based on the Joanna Briggs Institute data extraction template and aligned with the review objectives. Two authors independently extracted data, and disagreements were resolved through discussion. Studies were classified as diagnostic-performance studies if they evaluated a VFI-derived parameter as an index test against a defined reference standard and reported diagnostic-performance metrics including sensitivity, specificity, accuracy, and area under the curve. Diagnostic-performance studies were identified based on methodological criteria. Their clinical target was charted separately as stenosis grading, plaque vulnerability, symptomatic status, or stroke-risk/culprit-plaque assessment [28]. Technical studies were defined as those focused on the development or validation of VFI techniques rather than clinical application.

### 2.6. Critical Appraisal

No formal risk-of-bias appraisal was conducted, and no studies were excluded based on methodological quality, consistent with scoping review methodology [24,25,27]. This scoping review maps the evidence base rather than synthesizing effect sizes or pooled estimates. Methodological and reporting characteristics were extracted for all included studies to describe the evidence landscape. For studies reporting diagnostic-performance metrics, we extracted key quality indicators: patient selection; index test (blinding, prespecified thresholds); reference standard (appropriateness, blinding); flow and timing; sample size; Standards for Reporting of Diagnostic Accuracy (STARD) adherence [29]; ethics approval; and trial registration. This assessment mapped methodological rigor and reporting quality without excluding or weighting findings.

### 2.7. Synthesis of Results

**Figure 1:**
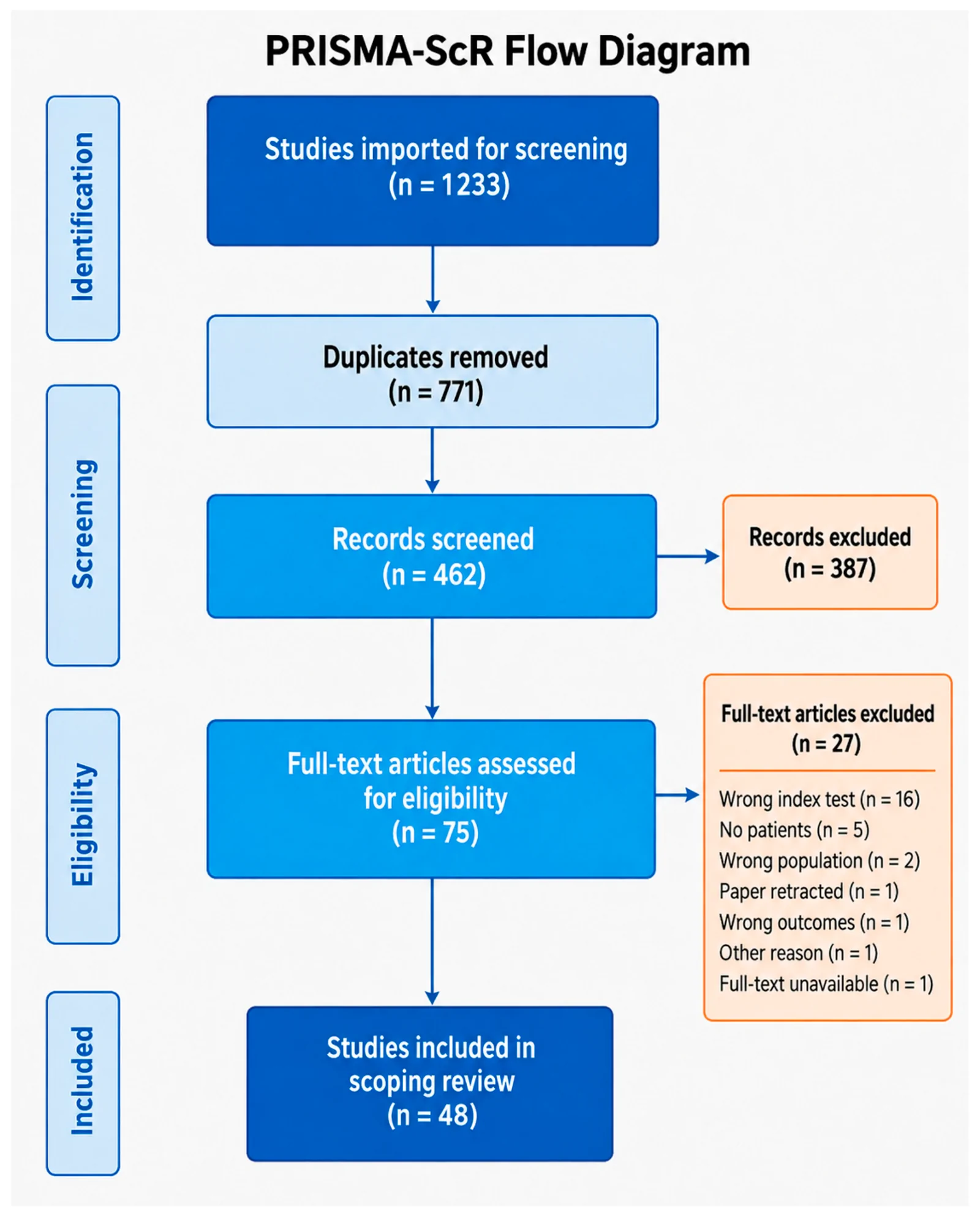
Flow of the screening and selection process.

Extracted data were synthesized using descriptive numerical analysis and narrative thematic synthesis and organized according to the review objectives. Descriptive statistics summarized temporal and geographic characteristics, study design, population, methodological features, VFI technology, and reference standards. Results were presented using frequencies, percentages, ranges, medians, and means as appropriate. No meta-analysis or pooled quantitative synthesis was conducted, consistent with scoping review methodology and anticipated heterogeneity. Narrative synthesis characterized VFI applications, identified VFI-derived parameters and their clinical relevance, examined diagnostic performance and validation approaches, and mapped research gaps and future directions. Results were presented using a PRISMA-ScR flow diagram (Figure 1), summary tables, figures, and narrative synthesis organized by objectives.

## 3. Results

### 3.1. Selection and Characteristics of Studies

The database searches yielded 462 records. Records were screened by title and abstract. Full-text articles were assessed for eligibility, and 48 sources of evidence [6,8,11,23,30,31,32,33,34,35,36,37,38,39,40,41,42,43,44,45,46,47,48,49,50,51,52,53,54,55,56,57,58,59,60,61,62,63,64,65,66,67,68,69,70,71,72,73] met the inclusion criteria and were included in the review. These comprised 42 studies with results and six trial registrations without reported results [49,55,64,65,72,73]. Of these, 40 were original studies, summarized in Table 1 and Table 2, and two articles were reviews [8,52]. The study selection process is illustrated in Figure 1. The majority of studies were published as full peer-reviewed journal articles (*n* = 32) [8,11,23,30,32,34,35,37,38,40,41,42,43,44,45,46,47,50,51,52,53,54,56,58,59,60,62,66,67,68,70,71], with six conference proceedings [6,31,48,57,63,69] and four conference abstracts [33,36,39,61].

A majority of the studies were published after 2023. Research was geographically concentrated in China and Europe. Two studies were published in languages other than English (one in Russian [59] and one in Chinese [58]). Study designs were predominantly cross-sectional (*n* = 20) and technical validation studies (*n* = 13), whereas quasi-experimental studies (*n* = 4), reviews (*n* = 2), case–control studies (*n* = 2), and case reports (*n* = 1) were rarer.

Thirty-nine studies reported 2353 total participants; one abstract did not report a sample size. Stenosis was reported among 2089 participants, including symptomatic (*n* = 515), asymptomatic (*n* = 929), and unknown (*n* = 645). Studies had predominantly small sample sizes (median 31, IQR 10.5–57; range 1–476). Among participants with reported sex, 70% were male. Populations included the full stenosis spectrum, including plaques. The assessment of CAS was highly heterogeneous across imaging modalities, including ultrasound, CT, DSA, and MRI, and in 14 studies, the imaging modality was not reported. The North American Symptomatic Carotid Endarterectomy Trial (NASCET) assessment for CAS grading [74] was the most commonly used grading system (*n* = 14); the European Carotid Surgery Trial (ECST) [75] grading system was used in six studies, and the grading methodology was not reported in 13 studies.

### 3.2. VFI Technology and Study Applications

Equipment information was reported in 32 studies, with clustering among certain commercial platforms. Mindray was the most frequently reported manufacturer (*n* = 17), followed by Verasonics (*n* = 9) and SuperSonic Imagine (*n* = 5). Other manufacturers included Hitachi and BK Medical (*n* = 2 each), as well as Ultrasonix, Esaote, and CSS (*n* = 1 each). The manufacturer was not reported in two studies. The VFI technique nomenclature was heterogeneous. Wall shear stress (WSS) was the most frequently investigated VFI-derived parameter (*n* = 25), with reporting methods varying across studies (mean, maximum, or both). Other parameters included turbulence indices (*n* = 11), velocity vectors (*n* = 14), and the oscillatory shear index (*n* = 8).

Study purposes varied, with plaque vulnerability/rupture prediction as the largest clinical application category (*n* =11), while stenosis degree assessment accounted for less than one-fifth of studies (*n* = 7). Reference standards varied across histology, advanced imaging modalities, clinical outcomes, and ultrasound-based methods, while 10 studies were proof-of-concept without external validation. Eight clinical studies were identified as diagnostic-performance studies [11,37,40,45,51,54,67,71] (Table 3). The studies were published since 2022 and predominantly assessed plaque vulnerability/stroke risk (*n* = 6), with three studies assessing stenosis severity [40,45,71]. Seven of the eight studies used Mindray platforms, and all were single-center. None of the studies employed prespecified thresholds. One study reported trial registration [37]. No studies reported adherence to STARD guidelines [29]. Across the full evidence base, cross-sectional and technical validation designs predominated, and sample sizes were generally small. Among the diagnostic-performance studies, one explicitly reported no blinding, while five did not clearly report their blinding status.

## 4. Discussion

This review identified a heterogeneous and predominantly early-stage evidence base, characterized by small cross-sectional and technical studies, variation in VFI implementation, and diverse clinical objectives. These characteristics limit the overall strength and comparability of the available evidence. Despite this heterogeneity, a consistent pattern emerged: WSS was the most frequently studied VFI-derived parameter, primarily to evaluate plaque vulnerability. This focus has become more pronounced in recent years, with larger clinical studies increasingly addressing applications related to vulnerability and symptoms, whereas stenosis grading remains less well studied. However, the larger clinical literature on WSS does not establish it as the most reproducible or clinically meaningful parameter. Among the diagnostic-performance studies, higher or peak WSS was generally associated with greater stenosis severity, symptomatic status, or plaque vulnerability. However, WSS directionality depended on anatomical and clinical context. Higher WSS was reported in patients with recent ipsilateral ischemic stroke and at or proximal to more severe and symptomatic stenoses [34,44]. In contrast, lower WSS was observed in downstream plaque regions and in association with greater intima media thickness [35,56]. Vulnerable plaques have also been reported to exhibit lower mean WSS upstream but higher maximum WSS at the stenosis peak [11]. This coexistence of lower and higher WSS around the same lesion is consistent with the spatial heterogeneity of shear around atherosclerotic stenoses [76]. Thus, WSS should be interpreted according to sampling location, lesion geometry, cardiac timing, and clinical endpoint rather than as a uniform high- or low-WSS marker of plaque vulnerability. Velocity-based measures were more often evaluated in technical validation studies, whereas turbulence indices and the OSI were generally assessed alongside WSS or within multimarker models, limiting assessment of their independent contribution.

Eight studies were classified as diagnostic-performance studies, exhibiting methodological characteristics common in early diagnostic test evaluation: none employed prespecified VFI thresholds; one study explicitly reported that index-test interpretation was not blinded; five did not clearly report blinding status; two reported blinding; and none reported conformance to the STARD guidelines. Together, these methodological and reporting limitations restrict assessment of bias and preclude firm conclusions regarding the overall strength of the diagnostic evidence. Current research supports the technical feasibility of VFI, but evidence of clinical utility remains preliminary. Despite technical feasibility, clinical translation should be considered separately for stenosis grading and plaque-vulnerability assessment. For stenosis grading, duplex ultrasound, CTA, MRA, and DSA are established within treatment pathways based primarily on stenosis severity and symptom status [1,77]. However, conventional Doppler is angle-dependent, whereas CTA, MRA, and DSA are generally more resource-intensive; CTA and DSA involve radiation and contrast administration, and DSA is invasive [4,5,7].

VFI offers angle-independent, radiation-free visualization of complex flow. Although VFI yields lower velocities than Doppler, comparative common-carotid studies have found closer agreement with phase-contrast MRI, indicating that lower values do not necessarily imply inferior accuracy [78,79]. Nevertheless, VFI-specific thresholds require validation against established reference standards. VFI has not yet demonstrated superior stenosis grading or altered clinical management. Most VFI studies instead evaluated secondary hemodynamic markers of plaque vulnerability. These may complement established vulnerability markers, although their incremental clinical value remains unproven. Such markers were not primary treatment-allocation criteria in recent large trials [1,80,81]. VFI should therefore currently be regarded as a potential complementary technique whose role in clinical decision-making remains to be established.

A major barrier to interpretation and clinical translation of VFI-derived evidence is the lack of standardized acquisition protocols and measurement approaches across studies. Table 3 further illustrates variation in anatomical sampling sites, cardiac-cycle selection, averaging methods, and the number of repeated measurements. Diagnostic-performance evidence was concentrated in WSS-containing models. However, the studies differed substantially in clinical targets, reference standards, sampling sites, cardiac timing, WSS definitions, data-derived thresholds, and multimarker composition. These differences limit direct comparison and attribution of reported performance to WSS alone and mean that thresholds and performance estimates cannot be assumed to transfer between studies. Although good intra- or interobserver agreement was observed within individual study settings [34,51,53,54,67], substantial variation in acquisition protocols, anatomical sampling sites, cardiac timing, parameter definitions, and analytical approaches limits the generalizability of these reproducibility findings to other study settings, centers, and imaging platforms. Direct comparisons with conventional Doppler remain limited, although they support technical feasibility; incremental value beyond established clinical or imaging assessment has not been externally validated.

VFI encompasses a variety of techniques rather than a single standardized implementation. Parameters derived from different commercial and research platforms should therefore not be assumed to be interchangeable, and comparability across manufacturers could not be assessed because seven of eight diagnostic-performance studies used Mindray V-Flow systems. Evidence regarding operator dependence, training requirements, reproducibility, workflow implications, and equipment availability remains incompletely characterized, limiting assessment of readiness for broad routine implementation.

The methodological limitations and knowledge gaps identified in this review define several priorities for future research. First, standardization of VFI acquisition, parameter definitions, and reporting is needed. Second, multicenter reproducibility studies should assess inter- and intraobserver reproducibility and validate prespecified thresholds. Third, cross-platform validation studies should compare VFI-derived measurements to establish whether measurements are interchangeable. Fourth, for applications in CAS, derivation and external validation of VFI-specific thresholds against established imaging reference standards are required. Fifth, for plaque vulnerability and stroke-risk applications, prospective multicenter outcome studies should evaluate whether VFI-derived parameters provide incremental predictive value beyond stenosis degree and symptom status. Sixth, studies reporting diagnostic-performance metrics should adhere to the STARD guidelines, including the prespecification of thresholds, the blinding of index test and reference standard interpretation, and prospective trial registration; clinical-impact trials should follow only after reproducibility and diagnostic validity have been established.

Although no language restrictions were imposed and internationally indexed non-English studies were eligible, Chinese domestic databases such as CNKI and Wanfang were not systematically searched. This represents an important limitation because a substantial proportion of the included studies originated from China, and relevant Chinese-language studies indexed only in domestic databases may therefore have been missed. Consequently, the present review may underestimate the volume of Chinese evidence and may not fully capture regional developments in VFI applications. The geographic concentration of studies in China and Europe may limit the generalizability of the findings to other healthcare settings and populations. Conference abstracts were included for descriptive evidence mapping; their limited methodological reporting restricted validity assessment, and none were classified as diagnostic-performance studies. No formal risk-of-bias or certainty-of-evidence assessment was undertaken. Instead, the methodological maturity of the evidence base was appraised qualitatively using the extracted study-design and reporting characteristics. Adherence to established scoping-review methodology was a strength, as this approach was better suited to the anticipated heterogeneity in study designs, VFI implementations, and outcome measures.

## 5. Conclusions

This scoping review identified 48 studies and trial registrations evaluating VFI in the assessment of CAS. The current evidence base is limited, methodologically heterogeneous, and largely composed of early cross-sectional studies focusing on feasibility and hemodynamic characterization rather than diagnostic validation. Key gaps include a lack of standardized acquisition and analysis protocols, limited diagnostic-performance data, the absence of validated VFI-specific thresholds against reference standards, and insufficient data on inter- and intra-observer reproducibility. VFI shows potential as an additional tool but currently remains an investigational technique requiring rigorous standardization and further validation before routine clinical implementation for carotid stenosis assessment.

## Figures and Tables

**Table 1:** Studies with WSS or WSR. Abbreviations: sample size (n), article (art.), conference (conf.), abstract (abstr.), cross-sectional (cross-sec.), case–control (case–ctrl.), technical study (tech.), quasi-experimental design (quasi), turbulence (Tur), oscillatory shear index (OSI), proof of concept (POC).

Authors	Year	Pub. Type	Design	n	Manufacturer	VFI Param	Study Purpose	Key Findings
Goudot et al. [33]	2019	Conf. abstr.	Cross-sec.	25	SuperSonic Imagine	WSS	General hemodynamic	Ultrafast VFI enabled direct WSS measurement along carotid plaques, with peak values at the plaque summit. Complemented conventional Doppler for identifying hemodynamically vulnerable areas.
Goudot et al. [61]	2019	Conf. abstr.	Cross-sec.	33	SuperSonic Imagine	WSS	CAS degree	VFI enabled direct local WSS measurement along carotid plaques. Provided better hemodynamic characterization than conventional Doppler for plaque vulnerability assessment.
Karageorgos et al. [48]	2019	Conf. art.	Tech.	1	Verasonics	Velocity, WSS	Tech./POC	Simultaneous pulse wave and vector flow imaging enabled combined assessment of arterial stiffness and WSS. Feasibility demonstrated in phantoms and one patient; broader validation is needed.
Saito et al. [35]	2020	Full art.	Cross-sec.	49	Hitachi	WSS	General hemodynamic	VFM Vascular enabled quantitative WSS measurement in the carotid artery. WSS correlated inversely with age and IMT, suggesting potential as a biomarker for early atherosclerosis.
Goudot et al. [50]	2020	Full art.	Case report	1	SuperSonic Imagine	WSS	Plaque vulnerability	Multimodal ultrafast ultrasound provided complementary WSS and stiffness biomarkers in a single patient. Supports individualized plaque vulnerability assessment beyond conventional B-mode.
Perrot et al. [68]	2020	Full art.	Tech.	25	Verasonics	WSR	Tech./POC	Vector flow imaging was feasible in vivo, showing larger diameters, more backflow, and lower WSR in diseased vessels.
Goudot et al. [36]	2021	Conf. abstr.	Cross-sec.	N/A	N/A	WSS	Plaque vulnerability	Combined SWE stiffness and WSS identified histologically vulnerable carotid plaques. Single-modality assessment was insufficient; multiparameter scoring is preferred.
Qiu et al. [47]	2021	Full art.	Cross-sec.	51	Mindray	WSS	CAS degree	V Flow visualized and quantified complex flow patterns in moderate and severe CAS. WSS measurements reflected stenosis severity and may support clinical management.
Zhang Huizhen et al. [58]	2021	Full art.	Case–ctrl.	120	Mindray	WSS	General hemodynamic	Lower WSS and higher IMT were found in sarcopenia patients compared to controls. VFI may detect early hemodynamic changes associated with cardiovascular risk in this population.
Goudot et al. [62]	2021	Full art.	Cross-sec.	33	SuperSonic Imagine	WSS, Tur	Tech./POC	WSS measurement characterized hemodynamic conditions along carotid plaques.
Wang et al. [39]	2022	Conf. abstr.	Cross-sec.	54	Mindray	WSS	CAS degree	V Flow WSS measurement differentiated severe from moderate carotid stenosis with acceptable diagnostic accuracy versus DSA.
Goudot et al. [37]	2022	Full art.	Cross-sec.	46	SuperSonic Imagine	WSS	Plaque vulnerability	Combined SWE and WSS identified histologically vulnerable carotid plaques with moderate accuracy. Neither modality alone was sufficient; combined analysis is recommended.
Karageorgos et al. [32]	2023	Full art.	Tech.	31	Verasonics	WSS	Plaque vulnerability	Automated WSS estimation using k-means clustering showed feasibility in phantoms and in vivo. Removes parabolic flow assumption; broader clinical validation is pending.
Qiu et al. [40]	2023	Full art.	Cross-sec.	88	Mindray	WSS	CAS degree/Plaque vulnerability	V Flow detected hemodynamic differences across stenosis severity grades. Proposed as a non-invasive preoperative assessment tool for symptomatic CAS.
Sandrikov et al. [59]	2023	Full art.	Quasi	53	Mindray	WSS, OSI, Tur	General hemodynamic	WSS normalized after carotid endarterectomy, reflecting restored hemodynamics. Vector flow mapping was sensitive to surgical outcome and may support post-operative monitoring.
Zhao et al. [34]	2024	Full art.	Cross-sec.	352	Mindray	WSS, OSI, Tur	Plaque vulnerability	WSS, OSI, and turbulence index from V Flow were associated with ischemic stroke risk in mild stenosis. Downstream plaque surface parameters may serve as hemodynamic stroke biomarkers.
Hong et al. [45]	2024	Full art.	Cross-sec.	54	Mindray	WSS, Tur	CAS degree	Combined WSS and turbulence index improved diagnostic accuracy for CAS over either parameter alone. Proposed as non-invasive dual-parameter tools for stroke-risk stratification.
Xiaoyong et al. [44]	2024	Full art.	Quasi	28	Mindray	WSS	General hemodynamic	WSS decreased at stenotic segments after successful carotid stenting. V Flow may support pre- and post-operative hemodynamic evaluation of carotid artery stenting.
Zhang et al. [54]	2024	Full art.	Cross-sec.	140	Mindray	WSS	Plaque vulnerability	Elevated WSS at the plaque’s thickest point was identified as a potential indicator of intra-plaque neovascularization. May support non-invasive prediction of carotid plaque vulnerability.
Chen et al. [56]	2024	Full art.	Cross-sec.	90	Mindray	WSS, OSI, Tur	General hemodynamic	In low-grade CAS, WSS was lower and flow disturbance was greater downstream than upstream of the plaque. WSS did not differ significantly between symptomatic and asymptomatic patients.
Zhao et al. [11]	2025	Full art.	Cross-sec.	41	Mindray	WSS, OSI, Tur	Plaque vulnerability	WSS and turbulence index correlated with plaque composition in advanced CAS. Combined VFI and conventional ultrasound may improve vulnerability assessment and guide treatment.
Zhang et al. [51]	2025	Full art.	Cross-sec.	476	Mindray	WSS, OSI, Tur	Plaque vulnerability	Multimodal ultrasonography distinguished culprit from non-culprit plaques in mild stenosis. Peak WSS and IPN classification were independent discriminators of plaque vulnerability.
Zhao et al. [53]	2025	Full art.	Case–ctrl.	122	Mindray	WSS, OSI, Tur	Plaque vulnerability	Downstream turbulence index and reduced elasticity independently predicted ipsilateral stroke in mild stenosis. Combined VFI and SWE outperformed single-modality assessment for stroke risk.
Shi et al. [67]	2026	Full art.	Cross-sec.	160	Mindray	WSS, OSI, Tur	Plaque vulnerability	Symptomatic patients showed higher OSI/Tur and WSS, with combined clinical, imaging, and V-Flow analysis improving plaque discrimination.
Yaylı et al. [71]	2026	Full art.	Cross-sec.	90	Mindray	WSS, OSI, Tur	CAS degree	WSSmean best detected ≥70% carotid stenosis; RF-ASI reflected global stiffness, not plaque severity.

**Table 2:** Studies investigating VFI parameters other than WSS. Protocols and reviews are excluded. Please refer to Table 1 for abbreviations.

Authors	Year	Pub. Type	Design	n	Manufacturer	VFI Param	Study Purpose	Key Findings
Vilkomerson et al. [6]	2005	Conf. art.	Cross-sec.	17	CSS	Velocity	CAS degree	A low-cost CW Vector Doppler screening device showed high sensitivity and specificity for CAS. Short scan times and low cost support feasibility for population-level screening.
Xu et al. [69]	2009	Conf. art.	Tech.	9	Hitachi	Velocity	Tech./POC	Position sensor registration reduced vector Doppler misregistration and enabled VFI detection of reversed flow near bifurcations and plaques.
Ekroll et al. [42]	2014	Full art.	Tech.	12	Ultrasonix	Velocity	Tech./POC	Plane wave VFI with automated PW Doppler calibration supported PSV accuracy over conventional spectral Doppler. Reduces operator dependency in carotid stenosis grading.
Tortoli et al. [43]	2015	Full art.	Cross-sec.	23	Esaote	Velocity	General hemodynamic	Two VFI methods outperformed conventional spectral Doppler for PSV measurement in CAS. Reproducibility was comparable across methods, supporting clinical adoption of VFI.
Ekroll et al. [31]	2017	Conf. art.	Tech.	2	Not reported	Velocity	Tech./POC	Tissue-adaptive clutter filters reduced velocity estimation bias in VFI. Performance matched SVD-based filtering, supporting more quantitative use of vector flow imaging.
Avdal et al. [41]	2017	Full art.	Tech.	2	Verasonics	Velocity	Tech./POC	Combined VFI and tracking Doppler improved peak velocity accuracy over conventional PW Doppler in CAS.
Brandt et al. [57]	2019	Conf. art.	Quasi	5	BK Medical	Vector concentration	Tech./POC	Vector concentration correlated with stenosis degree and improved after stenting. Proposed as a non-invasive alternative to DSA for stenosis assessment and treatment monitoring.
Karageorgos et al. [63]	2019	Conf. art.	Tech.	3	Verasonics	Velocity	Tech./POC	Adaptive PWI and vector Doppler mapped plaque stiffness and blood flow in vivo, revealing higher compliance at the stenosis confirmed by histology.
Saris et al. [66]	2019	Full art.	Tech.	5	Verasonics	Velocity	Tech./POC	Adaptive velocity compounding visualized complex carotid flow, with higher complexity in diseased vessels and post-stenotic reversed flow.
Brandt et al. [38]	2020	Full art.	Quasi	10	BK Medical	Vector concentration	CAS degree	Vector concentration correlated with stenosis degree and normalized after stenting, outperforming PSV. May offer a more reliable non-invasive measure for stenosis grading.
Karageorgos et al. [70]	2021	Full art.	Tech.	11	Verasonics	Velocity	Tech./POC	Cross-correlation VFI showed lower bias than vector Doppler. In vivo, compliance correlated with peak flow velocity and decreased with age, demonstrating feasibility in an atherosclerotic carotid.
Poloni et al. [60]	2023	Full art.	Tech.	6	Mindray	Velocity	General hemodynamic	HiFR-VFI showed strong agreement with MRI and CFD for velocity magnitude and direction in carotid bifurcations. Validated for detecting laminar flow and recirculation zones.
Dong et al. [46]	2024	Full art.	Cross-sec.	60	Mindray	Tur	Plaque vulnerability	Turbulence index from high-frame-rate VFI-quantified flow disturbance across stenosis grades. Proposed as a hemodynamic indicator for atherosclerotic risk stratification.
de Bakker et al. [23]	2025	Full art.	Tech.	14	Verasonics	Velocity	Tech./POC	Cascaded plane wave imaging improved blood SNR and velocity estimate reliability over conventional plane wave methods. Feasibility demonstrated in vivo; clinical validation is pending.
Haslund et al. [30]	2025	Full art.	Tech.	11	Verasonics	Pressure gradients	Tech./POC	Synthetic aperture ultrasound enabled reproducible non-invasive carotid pressure gradient estimation. Elevated values in a stenosis patient suggest potential for non-invasive hemodynamic grading.

**Table 3:** Diagnostic-performance studies, also presented in Table 1 and Table 2. All studies used Mindray V-Flow systems except Goudot et al. [37], who used a SuperSonic Imagine platform. WSSmean^S^, spatially averaged WSS; WSSmean^T^, temporally averaged WSS over one cardiac cycle; WSSmean^U^, mean WSS with unclear averaging dimension; WSS^U^, WSS with unspecified summary statistics. Classification of WSSmean was based on the averaging dimension explicitly reported in each study; when this was not specified, it was classified as WSSmean^U^. AUC, area under the receiver operating characteristic curve. All reported cut-offs were derived from the study data; none were prespecified.

Study	Clinical Target	n	Ref. Standard	Hemodynamic Parameters	WSS Method	Performance	Blinding
Goudot et al. [37]	Plaque vuln.	46	Histology	WSSmean^S^	Five plaque-surface ROIs; systolic frame with maximal WSS.	WSS alone: non-significant. Multimodal score including WSS > 1.74 Pa: cut-off ≥ 3; AUC 0.85; sensitivity 80%; specificity 78%.	No
Hong et al. [45]	Stenosis grading	54	B-mode ultrasound	WSSmean^T^; WSSmax; time-averaged Tur	Pre-, intra-, and post-stenotic sites; one cardiac cycle; three repeated measurements.	Tur > 22.98%: AUC 0.799; sensitivity 79.2%; specificity 83.3%. WSSmean > 2.647 Pa: AUC 0.840; sensitivity 87.5%; specificity 86.7%. Combined model: AUC 0.899; sensitivity 91.7%; specificity 80.0%.	Unclear
Qiu et al. [40]	Symptomatic status Stenosis grading	88	Clinical History CTA/DSA	Systolic WSS^U^	Stenotic segment during systole; WSS statistic unspecified.	Symptoms, WSS > 0.78 Pa: AUC 0.79; sensitivity 87.1%; specificity 69.6%. Severe stenosis, WSS > 0.90 Pa: AUC 0.90; sensitivity 86.2%; specificity 83.1%. Combined model: AUC 0.966; sensitivity 89.7%; specificity 93.2%.	Unclear
Shi et al. [67]	Plaque vuln.	160	Clinical history	WSSmean^S^; WSSmax; OSI; time-averaged turbulence intensity	Proximal, middle, and distal segments; ≥1 cardiac cycle acquired; temporal aggregation unreported.	Hemodynamic model: AUC 0.763. Clinical + Plaque-RADS + hemodynamic model: AUC 0.826. Other metrics were not reported.	Yes
Yaylı et al. [71]	Stenosis grading	90	CTA/MRA/DSA	WSSmean^U+S^; WSSmax; time-averaged turbulence	Proximal, peak, and distal segments at peak systole; two ROIs and three repeats averaged.	WSSmean > 2.25 Pa: AUC 0.96; sensitivity 96.8%; specificity 84.7%. WSSmax > 6.4 Pa: AUC 0.82; 74.2%; 74.6%. Tur > 29.5%: AUC 0.84; 80.6%; 74.6%. Time-averaged tur > 11.5%: AUC 0.82; 74.2%; 74.6%.	Unclear
Zhang et al. [51]	Plaque vuln.	476	Clinical history	WSSmean^U^; WSSmax; OSI; time-averaged turbulence	Five plaque sites; one cardiac cycle; WSS averaging dimension undefined.	Peak WSSmax > 2.93 Pa: AUC 0.768; sensitivity 85.7%; specificity 57.4%. Multivariable model, cut-off 0.22: AUC 0.856; sensitivity 85.7%; specificity 79.2%.	Yes
Zhang et al. [54]	Plaque vuln.	140	CEUS	WSSmean^U^; WSSmax	Three plaque sites; cardiac timing unreported; three repeats averaged.	WSSmax > 4.57 Pa: AUC 0.7762; sensitivity 74.03%; specificity 75.00%. WSSmean > 1.12 Pa: AUC 0.6973; sensitivity 63.64%; specificity 68.18%.	Unclear
Zhao et al. [11]	Plaque vuln.	41	Histology	WSSmean^U^; WSSmax; OSI; turbulence index	Upstream and stenosis peak; cardiac cycle identified; three repeats averaged; maximum-flow frame used for tur only.	Tur: AUC 0.843. Upstream WSSmean: 0.733. Peak WSSmax: 0.677. Peak WSSmean: 0.672. Plaque-area + tur model: cut-off 0.64; AUC 0.973; sensitivity 88%; specificity 100%.	Unclear

## Data Availability

No new data were created in this study. Data sharing is not applicable to this article.

## Abbreviations

The following abbreviations are used in this manuscript:

Abstr.	Abstract
art.	Article
AUC	Area under the curve
CAS	Carotid artery stenosis
case ctrl.	Case–control
Conf.	Conference
cross-sec.	Cross-sectional
CTA	Computed tomography angiography
DSA	Digital subtraction angiography
ECST	European Carotid Surgery Trial
MRA	Magnetic resonance angiography
n	Sample size
NASCET	North American Symptomatic Carotid Endarterectomy Trial
OSI	Oscillatory shear index
POC	Proof of concept
PRISMA-ScR	Preferred Reporting Items for Systematic Reviews and Meta-Analyses extension for Scoping Reviews
Quasi	Quasi-experimental design
Se	Sensitivity
Sp	Specificity
STARD	Standards for Reporting Diagnostic Accuracy Studies
Tech.	Technical study
Tur	Turbulence
VFI	Vector flow imaging
WSS	Wall shear stress

## Appendix A

**Table A1:** Preferred Reporting Items for Systematic reviews and Meta-Analyses extension for Scoping Reviews (PRISMA-ScR) checklist.

SECTION	ITEM	PRISMA-ScR CHECKLIST ITEM	REPORTED SECTION
**TITLE**			
Title	1	Identify the report as a scoping review.	Title
**ABSTRACT**			
Structured summary	2	Provide a structured summary that includes (as applicable): background, objectives, eligibility criteria, sources of evidence, charting methods, results, and conclusions that relate to the review questions and objectives.	Abstract
**INTRODUCTION**			
Rationale	3	Describe the rationale for the review in the context of what is already known. Explain why the review questions/objectives lend themselves to a scoping review approach.	Introduction
Objectives	4	Provide an explicit statement of the questions and objectives being addressed with reference to their key elements (e.g., population or participants, concepts, and context) or other relevant key elements used to conceptualize the review questions and/or objectives.	Introduction
**METHODS**			
Protocol and registration	5	Indicate whether a review protocol exists; state if and where it can be accessed (e.g., a Web address); and if available, provide registration information, including the registration number.	Method > Protocol
Eligibility criteria	6	Specify characteristics of the sources of evidence used as eligibility criteria (e.g., years considered, language, and publication status) and provide a rationale.	Method > Research Questions and Eligibility Criteria
Information sources	7	Describe all information sources in the search (e.g., databases with dates of coverage and contact with authors to identify additional sources), as well as the date the most recent search was executed.	Method > Information Sources and Search Strategy
Search	8	Present the full electronic search strategy for at least 1 database, including any limits used, such that it could be repeated.	Appendix C
Selection of sources of evidence †	9	State the process for selecting sources of evidence (i.e., screening and eligibility) included in the scoping review.	Method > Selection of Sources of Evidence and Data Charting
Data charting process ‡	10	Describe the methods of charting data from the included sources of evidence (e.g., calibrated forms or forms that have been tested by the team before their use and whether data charting was done independently or in duplicate) and any processes for obtaining and confirming data from investigators.	Method > Selection of Sources of Evidence and Data Charting
Data items	11	List and define all variables for which data were sought and any assumptions and simplifications made.	Protocol with adjustment in S2
Critical appraisal of individual sources of evidence §	12	If done, provide a rationale for conducting a critical appraisal of included sources of evidence; describe the methods used and how this information was used in any data synthesis (if appropriate).	Method > Critical Appraisal
Synthesis of results	13	Describe the methods of handling and summarizing the data that were charted.	Method > Synthesis of Results
**RESULTS**			
Selection of sources of evidence	14	Give numbers of sources of evidence screened, assessed for eligibility, and included in the review, with reasons for exclusions at each stage, ideally using a flow diagram.	Results > Selection and Characteristics of Studie Method > Figure 1 in Section 2.7
Characteristics of sources of evidence	15	For each source of evidence, present characteristics for which data were charted and provide the citations.	Results > Selection and Characteristics of Studies and VFI Technology and Study Applications
Critical appraisal within sources of evidence	16	If done, present data on critical appraisal of included sources of evidence (see item 12).	Results > VFI Technology and Study Applications
Results of individual sources of evidence	17	For each included source of evidence, present the relevant data that were charted that relate to the review questions and objectives.	Results
Synthesis of results	18	Summarize and/or present the charting results as they relate to the review questions and objectives.	Results
**DISCUSSION**			
Summary of evidence	19	Summarize the main results (including an overview of concepts, themes, and types of evidence available), link to the review questions and objectives, and consider the relevance to key groups.	Discussion
Limitations	20	Discuss the limitations of the scoping review process.	Discussion
Conclusions	21	Provide a general interpretation of the results with respect to the review questions and objectives, as well as potential implications and/or next steps.	Discussion
**FUNDING**			
Funding	22	Describe sources of funding for the included sources of evidence, as well as sources of funding for the scoping review. Describe the role of the funders of the scoping review.	Funding and Disclosures

## Appendix B

Protocol Deviations

Protocol Registration: OSF (https://osf.io/68ua7 (accessed on 20 August 2026); DOI: 10.17605/OSF.IO/68UA7)

This scoping review was conducted according to the registered protocol. All 40 protocol-specified data elements were captured. All core methodological elements were implemented as specified.

1. Data Extraction Platform

A custom relational database was used for data charting and extraction, whereas Covidence was used for title/abstract and full-text screening. Scoping reviews require flexible evidence mapping across heterogeneous study types; Covidence is optimized for systematic reviews with meta-analysis. All protocol elements were captured. Dual independent extraction was maintained.

2. Translation Method

AI-assisted translation was used for one Russian-language and one Chinese-language study. All extracted methodological details, numerical results, and table values used in the review were verified against the original-language full texts by reviewers proficient in Russian and Chinese, respectively. The previous abstract-level cross-verification was not used as validation of the full-text extraction.

3. Additional Elements and Categorizations

Five descriptive elements were added during pilot testing: publication type, equipment manufacturer, VFI technique nomenclature, WSS type specification, and study categorization (clinical/technical). Post-extraction categorizations were created for synthesis: study purposes (plaque vulnerability, technical validation, hemodynamic assessment, stenosis assessment, review) and reference standards (grouped by modality).

4. Extended search strategy

The original search strategy was changed to include studies with carotid plaques and other techniques that could be related to VFI.

## Appendix C

Search strings

PubMed (via NCBI)

(“Carotid Stenosis”[MeSH Terms] OR “Carotid Artery Diseases”[MeSH Terms] OR stenos*[Title/Abstract] OR “vulnerable plaque”[Title/Abstract] OR “carotid artery disease”[Title/Abstract] OR “carotid atherosclerosis”[Title/Abstract] OR (carotid[Title/Abstract] AND plaque*[Title/Abstract])) AND (“vector flow”[Title/Abstract] OR “vector doppler”[Title/Abstract] OR VFI[Title/Abstract] OR “flow vector”[Title/Abstract] OR “vector imag*”[Title/Abstract] OR “vector velocity”[Title/Abstract] OR “V-Flow”[Title/Abstract] OR “ultrafast ultrasound”[Title/Abstract] OR “ultrafast doppler”[Title/Abstract] OR “ultrafast imag*”[Title/Abstract] OR “synthetic aperture”[Title/Abstract] OR “high-frame-rate”[Title/Abstract] OR “high frame rate”[Title/Abstract] OR HiFR[Title/Abstract] OR “HiFR-VFI”[Title/Abstract])

Embase (via Ovid)

1. exp carotid stenosis/
2. “vulnerable plaque”.ab,fx,hw,kf,ot,ti,dq.
3. “carotid artery disease”.ab,fx,hw,kf,ot,ti,dq.
4. “carotid atherosclerosis”.ab,fx,hw,kf,ot,ti,dq.
5. (carotid adj3 plaque*).ab,fx,hw,kf,ot,ti,dq.
6. “stenos*”.ab,fx,hw,kf,ot,ti,dq.
7. “vector flow”.ab,fx,hw,kf,ot,ti,dq.
8. “vector doppler”.ab,fx,hw,kf,ot,ti,dq.
9. “VFI”.ab,fx,hw,kf,ot,ti,dq.
10. “flow vector”.ab,fx,hw,kf,ot,ti,dq.
11. “vector imag*”.ab,fx,hw,kf,ot,ti,dq.
12. “vector velocity”.ab,fx,hw,kf,ot,ti,dq.
13. “V-Flow”.ab,fx,hw,kf,ot,ti,dq.
14. “ultrafast ultrasound”.ab,fx,hw,kf,ot,ti,dq.
15. “ultrafast doppler”.ab,fx,hw,kf,ot,ti,dq.
16. “ultrafast imag*”.ab,fx,hw,kf,ot,ti,dq.
17. “synthetic aperture”.ab,fx,hw,kf,mf,ot,ti.
18. “high-frame-rate”.ab,fx,hw,kf,ot,ti,dq.
19. “high frame rate”.ab,fx,hw,kf,ot,ti,dq.
20. HiFR.ab,fx,hw,kf,ot,ti,dq.
21. 1 or 2 or 3 or 4 or 5 or 6
22. 7 or 8 or 9 or 10 or 11 or 12 or 13 or 14 or 15 or 16 or 17 or 18 or 19 or 20
23. 21 and 22

Scopus

TITLE-ABS-KEY(

(“Carotid Stenosis” OR stenos* OR “vulnerable plaque” OR “carotid artery disease”OR “carotid atherosclerosis” OR (carotid W/3 plaque*) )
AND
( “vector flow” OR “vector doppler” OR VFI OR “flow vector” OR (vector W/1 imag*)OR “vector velocity” OR “V-Flow” OR “ultrafast ultrasound” OR “ultrafast doppler”OR (ultrafast W/1 imag*) OR “synthetic aperture” OR “high-frame-rate” OR “high frame rate” OR “high-frame-rate vector flow imaging” OR “high frame rate vector flow imaging”
OR HiFR OR “HiFR-VFI” )
)

Web of Science (via Clarivate)

TS=(

(“Carotid Stenosis” OR stenos* OR “vulnerable plaque” OR “carotid artery disease” OR “carotid atherosclerosis” OR (carotid NEAR/3 plaque*)) AND (“vector flow” OR “vector doppler” OR VFI OR “flow vector” OR (vector NEAR/1 imag*) OR “vector velocity” OR “V-Flow” OR “ultrafast ultrasound” OR “ultrafast doppler” OR (ultrafast NEAR/1 imag*) OR “synthetic aperture” OR “high-frame-rate” OR “high frame rate” OR “high-frame-rate vector flow imaging” OR “high frame rate vector flow imaging” OR HiFR OR “HiFR-VFI”

)

)

IEEE Xplore

((((title:”Carotid Stenosis” OR “Abstract”:”Carotid Stenosis” OR “Document Title”:”stenos*” OR “Abstract”:”stenos*” OR “Document Title”:”vulnerable plaque” OR “Abstract”:”vulnerable plaque” OR “Document Title”:”carotid artery disease” OR “Abstract”:”carotid artery disease” OR “Document Title”:”carotid atherosclerosis” OR “Abstract”:”carotid atherosclerosis” OR “Document Title”:”carotid plaque” OR “Abstract”:”carotid plaque” OR “Document Title”:”carotid plaques” OR “Abstract”:”carotid plaques”) AND (title:”Vector Flow” OR “Abstract”:”Vector Flow” OR “Document Title”:”Vector Doppler” OR “Abstract”:”Vector Doppler” OR “Document Title”:”VFI” OR “Abstract”:”VFI” OR “Document Title”:”Flow vector” OR “Abstract”:”Flow vector” OR “Document Title”:”Vector imaging” OR “Abstract”:”Vector imaging” OR “Document Title”:”Vector velocity” OR “Abstract”:”Vector velocity” OR “Document Title”:”V-Flow” OR “Abstract”:”V-Flow” OR “Document Title”:”Ultrafast Ultrasound” OR “Abstract”:”Ultrafast Ultrasound” OR “Document Title”:”Ultrafast Doppler” OR “Abstract”:”Ultrafast Doppler” OR “Document Title”:”Ultrafast imaging” OR “Abstract”:”Ultrafast imaging” OR “Document Title”:”Synthetic aperture” OR “Abstract”:”Synthetic aperture” OR “Document Title”:”high-frame-rate” OR “Abstract”:”high-frame-rate” OR “Document Title”:”high frame rate” OR “Abstract”:”high frame rate” OR “Document Title”:”HiFR” OR “Abstract”:”HiFR” OR “Document Title”:”HiFR-VFI” OR “Abstract”:”HiFR-VFI”)) OR ((“MeSH Terms”:”Carotid Stenosis” OR “IEEE Terms”:”Carotid Stenosis” OR “Document Title”:”stenos*” OR “Abstract”:”stenos*”) AND (ieeeTerms:”Vector Flow Imaging” OR “Document Title”:”Vector Flow” OR “Abstract”:”Vector Flow” OR “Document Title”:”Vector Doppler” OR “Abstract”:”Vector Doppler” OR “Document Title”:”VFI” OR “Abstract”:”VFI” OR “Document Title”:”Flow vector” OR “Abstract”:”Flow vector” OR “Document Title”:”Vector imag*” OR “Abstract”:”Vector imag*” OR “Document Title”:”Vector velocity” OR “Abstract”:”Vector velocity” OR “Document Title”:”V-Flow” OR “Abstract”:”V-Flow” OR “Document Title”:”Ultrafast Ultrasound” OR “Abstract”:”Ultrafast Ultrasound” OR “Document Title”:”Ultrafast Doppler” OR “Abstract”:”Ultrafast Doppler” OR “Document Title”:”Ultrafast imag*” OR “Abstract”:”Ultrafast imag*” OR “Document Title”:”Synthetic aperture” OR “Abstract”:”Synthetic aperture”))))

ICTRP

(“Carotid Stenosis” OR “stenos*”) AND (“ vector flow” OR “vector Doppler” OR “VFI” OR “flow vector” OR “vector imag*” OR “vector velocity” OR “V-Flow” OR “ultrafast ultrasound” OR “ultrafast doppler” OR “ultrafast imag*” OR “ synthetic aperture”)

## References

[1] Naylor R., Rantner B., Ancetti S., de Borst G.J., De Carlo M., Halliday A., Kakkos S.K., Markus H.S., McCabe D.J.H., Sillesen H. (2023). Editor’s Choice—European Society for Vascular Surgery (ESVS) 2023 Clinical Practice Guidelines on the Management of Atherosclerotic Carotid and Vertebral Artery Disease. Eur. J. Vasc. Endovasc. Surg..

[2] Bonati L.H., Kakkos S., Berkefeld J., de Borst G.J., Bulbulia R., Halliday A., van Herzeele I., Koncar I., McCabe D.J., Lal A. (2021). European Stroke Organisation Guideline on Endarterectomy and Stenting for Carotid Artery Stenosis. Eur. Stroke J..

[3] Chambers B.R., Donnan G. (2005). Carotid endarterectomy for asymptomatic carotid stenosis. Cochrane Database Syst. Rev..

[4] Wardlaw J., Chappell F., Best J., Wartolowska K., Berry E. (2006). Non-Invasive Imaging Compared with Intra-Arterial Angiography in the Diagnosis of Symptomatic Carotid Stenosis: A Meta-Analysis. Lancet.

[5] Patel S.G. (2002). Outcome, Observer Reliability, and Patient Preferences If CTA, MRA, or Doppler Ultrasound Were Used, Individually or Together, Instead of Digital Subtraction Angiography before Carotid Endarterectomy. J. Neurol. Neurosurg. Psychiatry.

[6] Vilkomerson D., Chilipka T., Outcault R., Goldman K. (2005). An Instrument for Screening for Carotid Stensoses. Proceedings of the IEEE Ultrasonics Symposium, 2005.

[7] Cassola N., Baptista-Silva J.C.C., Nakano L.C.U., Flumignan C.D.Q., Sesso R., Vasconcelos V., Carvas Junior N., Flumignan R.L.G. (2022). Duplex ultrasound for diagnosing symptomatic carotid stenosis in the extracranial segments. Cochrane Database Syst. Rev..

[8] Hansen K.L., Nielsen M.B., Jensen J.A. (2017). Vector Velocity Estimation of Blood Flow—A New Application in Medical Ultrasound. Ultrasound.

[9] Jensen J.A., Nikolov S., Yu A.C.H., Garcia D. (2016). Ultrasound Vector Flow Imaging—Part I: Sequential Systems. IEEE Trans. Ultrason. Ferroelect. Freq. Contr..

[10] Baun J. (2021). Emerging Technology: Ultrasound Vector Flow Imaging—A Novel Approach to Arterial Hemodynamic Quantification. J. Diagn. Med. Sonogr..

[11] Zhao Y.-C., He Y.-M., Wang F., Xu M.-J., Zhang D., Wang D.-H., Zhang M. (2025). Blood Flow Turbulence Measured by High-Frame-Rate Vector Flow Imaging Conduced to Investigating Advanced Carotid Plaque Vulnerability. Ultrasound Med. Biol..

[12] Jensen J.A., Munk P. (1998). A New Method for Estimation of Velocity Vectors. IEEE Trans. Ultrason. Ferroelect. Freq. Contr..

[13] Jensen J.A. (2001). A New Estimator for Vector Velocity Estimation [Medical Ultrasonics]. IEEE Trans. Ultrason. Ferroelect. Freq. Contr..

[14] Trahey G.E., Allison J.W., Von Ramm O.T. (1987). Angle Independent Ultrasonic Detection of Blood Flow. IEEE Trans. Biomed. Eng..

[15] Montaldo G., Tanter M., Bercoff J., Benech N., Fink M. (2009). Coherent Plane-Wave Compounding for Very High Frame Rate Ultrasonography and Transient Elastography. IEEE Trans. Ultrason. Ferroelect. Freq. Contr..

[16] Sandrin L., Manneville S., Fink M. (2001). Ultrafast Two-Dimensional Ultrasonic Speckle Velocimetry: A Tool in Flow Imaging. Appl. Phys. Lett..

[17] Udesen J., Gran F., Hansen K., Jensen J.A., Thomsen C., Nielsen M.B. (2008). High Frame-Rate Blood Vector Velocity Imaging Using Plane Waves: Simulations and Preliminary Experiments. IEEE Trans. Ultrason. Ferroelect. Freq. Contr..

[18] Jensen A.J., Nikolov S.I. (2004). Directional Synthetic Aperture Flow Imaging. IEEE Trans. Ultrason. Ferroelect. Freq. Contr..

[19] Nikolov S.I., Jensen J.A. (2003). In-Vivo Synthetic Aperture Flow Imaging in Medical Ultrasound. IEEE Trans. Ultrason. Ferroelect. Freq. Contr..

[20] Fox M.D. (1978). Multiple Crossed-Beam Ultrasound Doppler Velocimetry. IEEE Trans. Son. Ultrason..

[21] Yiu B.Y.S., Lai S.S.M., Yu A.C.H. (2014). Vector Projectile Imaging: Time-Resolved Dynamic Visualization of Complex Flow Patterns. Ultrasound Med. Biol..

[22] Udesen J., Nielsen M.B., Nielsen K.R., Jensen J.A. (2007). Examples of In Vivo Blood Vector Velocity Estimation. Ultrasound Med. Biol..

[23] De Bakker J.M.K., Ruisch J., De Korte C.L., Saris A.E.C.M. (2025). Cascaded Plane Wave Ultrasound Velocity Vector Imaging: In Vivo Feasibility in Carotid Arteries. IEEE Trans. Ultrason. Ferroelect. Freq. Contr..

[24] Aromataris E., Lockwood C., Porritt K., Pilla B., Jordan Z. (2024). JBI Manual for Evidence Synthesis.

[25] Tricco A.C., Lillie E., Zarin W., O’Brien K.K., Colquhoun H., Levac D., Moher D., Peters M.D.J., Horsley T., Weeks L. (2018). PRISMA Extension for Scoping Reviews (PRISMA-ScR): Checklist and Explanation. Ann. Intern. Med..

[26] Arksey H., O’Malley L. (2005). Scoping Studies: Towards a Methodological Framework. Int. J. Soc. Res. Methodol..

[27] Levac D., Colquhoun H., O’Brien K.K. (2010). Scoping Studies: Advancing the Methodology. Implement. Sci..

[28] Leeflang M.M.G., Deeks J.J., Gatsonis C., Bossuyt P.M.M. (2008). Systematic Reviews of Diagnostic Test Accuracy. Ann. Intern. Med..

[29] Cohen J.F., Korevaar D.A., Altman D.G., Bruns D.E., Gatsonis C.A., Hooft L., Irwig L., Levine D., Reitsma J.B., De Vet H.C.W. (2016). STARD 2015 Guidelines for Reporting Diagnostic Accuracy Studies: Explanation and Elaboration. BMJ Open.

[30] Haslund L.E., Henriksen A.C., Yiu B.Y.S., Salari A., Traberg M.S., Jørgensen L.T., Tomov B.G., Nielsen M.B., Jensen J.A. (2025). Precision of in Vivo Pressure Gradient Estimations Using Synthetic Aperture Ultrasound. Ultrasonics.

[31] Ekroll I.K., Avdal J. (2017). Adaptive Clutter Filtering Based on Tissue Vector Velocities. Proceedings of the 2017 IEEE International Ultrasonics Symposium (IUS).

[32] Karageorgos G.M., Kemper P., Lee C., Weber R., Kwon N., Meshram N., Mobadersany N., Grondin J., Marshall R.S., Miller E.C. (2023). Adaptive Wall Shear Stress Imaging in Phantoms, Simulations and In Vivo. IEEE Trans. Biomed. Eng..

[33] Goudot G., Pedreira O., Poree J., Khider L., Mirault T., Julia P., Alsac J.M., Pernot M., Messas E. (2019). Assessment of Wall Shear Stress by Ultrafast Vector Flow Imaging in Carotid Atheromatous Stenosis. Arch. Cardiovasc. Dis. Suppl..

[34] Zhao M., Zhang L., Chen J., Gu S., Wu R., Jia C. (2024). Associations between Carotid Plaque Shape, Biomechanical Parameters, and Ischemic Stroke in Mild Carotid Stenosis with a Single Plaque. Ultrasonography.

[35] Saito K., Abe S., Kumamoto M., Uchihara Y., Tanaka A., Sugie K., Ihara M., Koga M., Yamagami H. (2020). Blood Flow Visualization and Wall Shear Stress Measurement of Carotid Arteries Using Vascular Vector Flow Mapping. Ultrasound Med. Biol..

[36] Goudot G., Sitruk J., Jimenez A., Khider L., Julia P., Alsac J.M., El Batti S., Bruneval P., Amemyia K., Pedreira O. (2021). Carotid Plaque Vulnerability Assessed by Combined Shear Wave Elastography and Ultrafast Doppler Compared to Histology: The UF-Plaque Study. Eur. Heart J..

[37] Goudot G., Sitruk J., Jimenez A., Julia P., Khider L., Alsac J.-M., El Batti S., Bruneval P., Amemyia K., Pedreira O. (2022). Carotid Plaque Vulnerability Assessed by Combined Shear Wave Elastography and Ultrafast Doppler Compared to Histology. Transl. Stroke Res..

[38] Brandt A.H., Nguyen T.-Q., Gutte H., Frederik Carlsen J., Moshavegh R., Jensen J.A., Bachmann Nielsen M., Hansen K.L. (2020). Carotid Stenosis Assessment with Vector Concentration before and after Stenting. Diagnostics.

[39] Wang W., Qiu Y., Yang D.-H., Zhang Q., Dong Y. (2022). Clinical Application of High-Frame Rate Vector Flow Imaging Technology in Evaluating Hemodynamic Changes of Moderate and Severe Carotid Stenosis. Insights Imaging.

[40] Qiu Y.-J., Cheng J., Zhang Q., Yang D.-H., Zuo D., Mao F., Liu L.-X., Dong Y., Cao S.-Q., Wang W.-P. (2023). Clinical Application of High-Frame-Rate Vector Flow Imaging in Evaluation of Carotid Atherosclerotic Stenosis. Diagnostics.

[41] Avdal J., Lovstakken L., Torp H., Ekroll I.K. (2017). Combined 2-D Vector Velocity Imaging and Tracking Doppler for Improved Vascular Blood Velocity Quantification. IEEE Trans. Ultrason. Ferroelectr. Freq. Control.

[42] Ekroll I.K., Dahl T., Torp H., Løvstakken L. (2014). Combined Vector Velocity and Spectral Doppler Imaging for Improved Imaging of Complex Blood Flow in the Carotid Arteries. Ultrasound Med. Biol..

[43] Tortoli P., Lenge M., Righi D., Ciuti G., Liebgott H., Ricci S. (2015). Comparison of Carotid Artery Blood Velocity Measurements by Vector and Standard Doppler Approaches. Ultrasound Med. Biol..

[44] Xiaoyong T., Yuping C., Wei H., Juan C., Feng Q., Zhuo L. (2024). Evaluafion of the Efficacy of Wall Shear Stress in Carotid Artery Stenting. Heliyon.

[45] Hong S., Dong Y., Gao W., Song D., Liu M., Li W., Du Y., Xu J., Dong F. (2024). Evaluation of Carotid Stenosis in a High-Stroke-Risk Population by Hemodynamic Dual-Parameters Based on Ultrasound Vector Flow Imaging. Brain Behav..

[46] Dong Y., Gao W., Hong S., Song D., Liu M., Du Y., Xu J., Dong F. (2024). Evaluation of Turbulence Index and Flow Pattern for Atherosclerotic Carotid Stenosis: A High-Frame-Rate Vector Flow Imaging Study. Ultrasound Med. Biol..

[47] Qiu Y., Dong Y., Mao F., Zhang Q., Yang D., Chen K., Shi S., Zuo D., Tian X., Yu L. (2021). High-Frame Rate Vector Flow Imaging Technique: Initial Application in Evaluating the Hemodynamic Changes of Carotid Stenosis Caused by Atherosclerosis. Front. Cardiovasc. Med..

[48] Karageorgos G.M., Zacharias Apostolakis I., Nauleau P., Gatti V., Weber R., Grondin J., Konofagou E.E. (2019). Imaging of Pulse Wave Propagation Coupled with Vector Flow and Wall Shear Stress Mapping in Atherosclerotic Plaque Phantoms and In Vivo. Proceedings of the 2019 IEEE International Ultrasonics Symposium (IUS).

[49] Radboud University Medical Center Improved Image Quality for Assessment of Carotid Artery Stenosis by Ultrafast Ultrasound FLOW Imaging. ClinicalTrials.gov 2023. https://clinicaltrials.gov/study/NCT06170580.

[50] Goudot G., Khider L., Pedreira O., Poree J., Julia P., Alsac J.-M., Amemiya K., Bruneval P., Messas E., Pernot M. (2020). Innovative Multiparametric Characterization of Carotid Plaque Vulnerability by Ultrasound. Front. Physiol..

[51] Zhang J., Wu X., Gao Y., Wang L. (2025). Morphological and Biomechanical Characteristics of Carotid Plaques with Mild Stenosis Using Ultrafast and High-Definition Ultrasonography. Eur. Radiol..

[52] David E., Martinelli O., Pacini P., Di Serafino M., Huang P., Dolcetti V., Del Gaudio G., Barr R.G., Renda M., Lucarelli G.T. (2023). New Technologies in the Assessment of Carotid Stenosis: Beyond the Color-Doppler Ultrasound-High Frame Rate Vector-Flow and 3D Arterial Analysis Ultrasound. Diagnostics.

[53] Zhao M., Chen J., Gu S., Hu H., Zhang L., Wu R., Jia C. (2025). Plaque Biomarkers from High-Frame Rate Vector Flow Imaging and Shear Wave Elastography in Mild Carotid Stenosis. Ultrasonography.

[54] Zhang X., Ding H., Ji X., Chen L., Huang P., Lin Z., Zhu J., Zhou S., Liu Z., Zhang M. (2024). Predicting Vulnerable Carotid Plaques by Detecting Wall Shear Stress Based on Ultrasonic Vector Flow Imaging. J. Vasc. Surg..

[55] Hospital Rijnstate Progression Assessment of Carotid Artery Stenosis by Ultrafast Ultrasound Flow Imaging. ClinicalTrials.gov 2022. https://clinicaltrials.gov/study/NCT05270005.

[56] Chen J., Zhang L., Gu S., Jia C., Wu R. (2024). Quantitative Evaluation Using Carotid Ultrasonography-Based High-Frame-Rate Vector Flow Imaging in Patients with Low Carotid Stenosis. Br. J. Radiol..

[57] Brandt A.H., Nguyen T.-Q., Gutte H., Holtmannspotter M., Moshavegh R., Jensen J.A., Nielsen M.B., Hansen K.L. (2019). Vector Concentration Used for Stenosis Assessment in the Carotid Artery Before and After Carotid Stenting. Proceedings of the 2019 IEEE International Ultrasonics Symposium (IUS).

[58] Zhang H., Lin N., Yang L., Chen Y., Tang L., Yu M. (2021). Vector Flow Imaging for Evaluation on Carotid Artery Changes in Patients with Sarcopenia. Chin. J. Med. Imaging Technol..

[59] Sandrikov V.A., Dutikova E.F., Gavrilenko A.V., Kulagina T.Y., Zhigulina O.A., Lebedeva E.Y. (2023). Vector Mapping of Intravascular Flows to Assess Wall Shear Stress in Patients with Internal Carotid Artery Stenosis. Angiol. Sosud. Khir..

[60] Poloni S., Bozzetto M., Du Y., Aiani L., Goddi A., Fiorina I., Remuzzi A. (2023). Velocity Vector Comparison between Vector Flow Imaging and Computational Fluid Dynamics in the Carotid Bifurcation. Ultrasonics.

[61] Goudot G., Khider L., Pedreira O., Poree J.M., Julia P., Alsac J.M., Mirault T., Pernot M., Messas E. (2019). Wall Shear Stress Measurement by Ultrafast Vector Flow Imaging for Atherosclerotic Carotid Stenosis. Eur. Heart J..

[62] Goudot G., Poree J., Pedreira O., Khider L., Julia P., Alsac J.-M., Laborie E., Mirault T., Tanter M., Messas E. (2021). Wall Shear Stress Measurement by Ultrafast Vector Flow Imaging forÂ Atherosclerotic Carotid Stenosis. Ultraschall Med..

[63] Karageorgos G.M., Apostolakis I.Z., Nauleau P., Gatti V., Weber R., Konofagou E.E. (2019). Atherosclerotic Plaque Mechanical Characterization Coupled with Vector Doppler Imaging in Atherosclerotic Carotid Arteries In-Vivo. Proceedings of the 2019 41st Annual International Conference of the IEEE Engineering in Medicine and Biology Society (EMBC).

[64] Intelligence Based Comprehensive Assessment System of Carotid Plaque Stability. ClinicalTrials.gov 2020. https://clinicaltrials.gov/study/NCT04544657.

[65] An Integrated System for the Assessment of Carotid Plaque Stability Based on the Artificial Intelligence. ClinicalTrials.gov 2021. https://clinicaltrials.gov/study/NCT04928547.

[66] Saris A.E.C.M., Hansen H.H.G., Fekkes S., Menssen J., Nillesen M.M., de Korte C.L. (2019). In Vivo Blood Velocity Vector Imaging Using Adaptive Velocity Compounding in the Carotid Artery Bifurcation. Ultrasound Med. Biol..

[67] Shi L., Ning Y., Wang Q., Fang Y., Yao T., Zhang J., Zheng K., Yan S., Li H., Shi Y. (2026). Multimodal Risk Stratification Clue of Carotid Bifurcation Plaques: A Cross-Sectional Observational Study of Vector Flow Imaging, Plaque-RADS, and Clinical Factors. Transl. Stroke Res..

[68] Perrot V., Ekroll I.K., Avdal J., Saxhaug L.M., Dalen H., Vray D., Lovstakken L., Liebgott H. (2021). Translation of Simultaneous Vessel Wall Motion and Vectorial Blood Flow Imaging in Healthy and Diseased Carotids to the Clinic: A Pilot Study. IEEE Trans. Ultrason. Ferroelect. Freq. Contr..

[69] Xu C., Beach K.W., Leotta D., Stuzman E., Kim Y. (2009). Reducing Registration Error in Cross-Beam Vector Doppler Imaging with Position Sensor. Proceedings of the 2009 Annual International Conference of the IEEE Engineering in Medicine and Biology Society.

[70] Karageorgos G.M., Apostolakis I.-Z., Nauleau P., Gatti V., Weber R., Kemper P., Konofagou E.E. (2021). Pulse Wave Imaging Coupled With Vector Flow Mapping: A Phantom, Simulation, and In Vivo Study. IEEE Trans. Ultrason. Ferroelectr. Freq. Control.

[71] Yaylı N., Kılıç A.C.K., Biçer B.K., Öncü F., Altıparmak T., Cerit M.N., Şendur H.N., Çağlayan H.Z.B., Akkan M.K., Kılıç H.K. (2026). Diagnostic Performance of Vector Flow Imaging–Derived Wall Shear Stress and Radiofrequency Echo-Tracking–Derived Arterial Stiffness Indices in Severe Carotid Stenosis. Ultrasonography.

[72] Plaque-RADS Combined With Vector Flow Imaging in Assessing Carotid Atherosclerotic Risk. ClinicalTrials.gov 2025. https://clinicaltrials.gov/study/NCT07178015.

[73] Blood Flow Evaluation After Carotid Surgical Treatment: Carotid Endarterectomy with Patch Repair Versus Eversion Technique. ClinicalTrials.gov 2025. https://clinicaltrials.gov/study/NCT06827509.

[74] North American Symptomatic Carotid Endarterectomy Trial Collaborators (1991). Beneficial Effect of Carotid Endarterectomy in Symptomatic Patients with High-Grade Carotid Stenosis. N. Engl. J. Med..

[75] (1991). MRC European Carotid Surgery Trial: Interim Results for Symptomatic Patients with Severe (70-99%) or with Mild (0-29%) Carotid Stenosis. European Carotid Surgery Trialists’ Collaborative Group. Lancet.

[76] Urschel K., Tauchi M., Achenbach S., Dietel B. (2021). Investigation of Wall Shear Stress in Cardiovascular Research and in Clinical Practice-From Bench to Bedside. Int. J. Mol. Sci..

[77] AbuRahma A.F., Avgerinos E.D., Chang R.W., Darling R.C., Duncan A.A., Forbes T.L., Malas M.B., Murad M.H., Perler B.A., Powell R.J. (2022). Society for Vascular Surgery Clinical Practice Guidelines for Management of Extracranial Cerebrovascular Disease. J. Vasc. Surg..

[78] Du Y., Ding H., He L., Yiu B.Y.S., Deng L., Yu A.C.H., Zhu L. (2022). Quantitative Blood Flow Measurements in the Common Carotid Artery: A Comparative Study of High-Frame-Rate Ultrasound Vector Flow Imaging, Pulsed Wave Doppler, and Phase Contrast Magnetic Resonance Imaging. Diagnostics.

[79] Brandt A.H., Hansen K.L., Ewertsen C., Holbek S., Olesen J.B., Moshavegh R., Thomsen C., Jensen J.A., Nielsen M.B. (2018). A Comparison Study of Vector Velocity, Spectral Doppler and Magnetic Resonance of Blood Flow in the Common Carotid Artery. Ultrasound Med. Biol..

[80] Brott T.G., Howard G., Lal B.K., Voeks J.H., Turan T.N., Roubin G.S., Lazar R.M., Brown R.D., Huston J., Edwards L.J. (2026). Medical Management and Revascularization for Asymptomatic Carotid Stenosis. N. Engl. J. Med..

[81] Donners S.J.A., Van Velzen T.J., Cheng S.F., Gregson J., Hazewinkel A.-D., Pizzini F.B., Emmer B.J., Simister R., Richards T., Lyrer P.A. (2025). Optimised Medical Therapy Alone versus Optimised Medical Therapy plus Revascularisation for Asymptomatic or Low-to-Intermediate Risk Symptomatic Carotid Stenosis (ECST-2): 2-Year Interim Results of a Multicentre Randomised Trial. Lancet Neurol..

